# Comparison between rosuvastatin and atorvastatin for the prevention of contrast-induced nephropathy in patients with STEMI undergoing primary percutaneous coronary intervention

**DOI:** 10.15171/jcvtr.2018.24

**Published:** 2018-09-24

**Authors:** Ata Firouzi, Ali Kazem Moussavi, Ahmad Mohebbi, Mohammad Javad Alemzadeh-Ansari, Reza Kiani, Hamid Reza Sanati, Bahram Mohebbi, Farshad Shakerian, Ali Zahedmehr, Mohammad Mostafa Ansari-Ramandi, Saeed Oni Heris, Bahar Ghaleshi, Fatemeh Ghorbani

**Affiliations:** Rajaie Cardiovascular Medical and Research Center, Iran University of Medical Sciences, Tehran, Iran

**Keywords:** Contrast-induced Nephropathy, Statin, Percutaneous coronary intervention

## Abstract

***Introduction:*** There is some controversy over the efficacy of statins for the prevention of contrastinduced nephropathy (CIN). There have also been reports on varying efficacies of different statins. Hence, in this study the efficacy of atorvastatin and rosuvastatin for the prevention of CIN was assessed.

***Methods:*** This single-blind randomized clinical trial was performed on 495 random patients with myocardial infarction with ST-segment elevation undergoing primary percutaneous coronary intervention (PCI) in a training referral hospital in 2015. Patients were randomly assigned to receive either atorvastatin 80 mg at admission and daily or rosuvastatin 40 mg at admission and daily. CIN was defined based on serum creatinine elevation after 48 hours from the PCI.

***Results:*** The incidence of CIN was observed in 63 patients (21.4%) After 48 hours from primary PCI. Of those, 17% (n = 50) were grade 1 CIN, while 4.4% (n = 13) were grade 2 CIN. There was no significant difference between rosuvastatin group compared with atorvastatin group, regarding the CIN grading (*P* = 0.14).

***Conclusion:*** Our results indicate that atorvastatin and rosuvastatin have similar efficacy for the prevention of CIN.

## Introduction

The leading cause of mortality worldwide, coronary artery disease is on the rise owing to higher sanitary levels, urbanization, and aging populations.^1-3^ Coronary artery disease is responsible for 6.4 deaths per 10000 Iranian population, and 35% of all mortalities are due to cardiac diseases.^4,5^ Coronary artery bypass grafting and percutaneous coronary interventions (PCI) are the principal revascularization approaches.^6-8^ Early PCI is the method of choice for myocardial infarction with ST elevation, and a shorter interval between event and hospital arrival can result in lower mortality rates.^7-9^

Coronary angiography is the most common heart procedure worldwide, and contrast-induced nephropathy (CIN) has shown increased rates in those undergoing this modality. CIN is commonly observed among patients undergoing primary PCI, even in those with a normal renal function.^10^ Chronic kidney alterations may affect those with previous renal insufficiency up to 12%; however, the symptoms are seen in less than 1%. CIN is an acute decreased renal function after an intravenous infusion of iodine contrast media, which is the third cause of hospital-acquired acute renal failure and is due to cardiac procedures in half of the cases.^11-13^ Furthermore, it may increase the risk of hemodialysis and death.^10^

CIN may be secondary to direct tubular toxicity, vasoconstriction, and oxidative stress.^14-16^ Statins (HMG-CoA reductase inhibitor) may lessen atherosclerosis, inflammation, endothelial dysfunction, and platelet hyperactivity.^15^ Good effects of statins such as atorvastatin and rosuvastatin on oxidative stress, nitric oxide synthesis, and endothelial function constitute some of the mechanisms responsible for the renoprotective effects in those with chronic kidney disease.^14^ Nevertheless, not only is there controversy surrounding the efficacy of statins for the prevention of CIN, but also there have been reports on varying efficacies of various statins.^15-21^ Accordingly, in this study the efficacy of atorvastatin and rosuvastatin for the prevention of CIN was assessed among patients undergoing primary PCI.

## Materials and Methods

This single-blind randomized clinical trial was performed on 302 random patients with myocardial infarction with ST-segment elevation undergoing primary PCI in a training referral hospital in 2015. The patients with known hypersensitivity to statins, those with cardiogenic shock status, pregnant and lactating females, and those who had received a contrast agent within the preceding week were excluded from the study.

The patients were randomly assigned to receive either atorvastatin (n = 150) or rosuvastatin (n = 152). Unfortunately, 7 patients died before 48 hours from presentation. Thus, 144 patients in atorvastatin group and 151 patients in rosuvastatin were evaluated.

Atorvastatin dose was 80 mg at admission and daily up to 48 hours later, and rosuvastatin dose was 40 mg at admission and daily up to 48 hours after the procedure. Before the PCI procedure, hemoglobin, lipid profile, baseline blood urea nitrogen (BUN), creatinine, and glomerular filtration rate (GFR) were assessed. The Mehran CIN-Risk score was calculated based on Mehran et al study.^22^ Thereafter, BUN, creatinine, and GFR were assessed for 48 hours.

The prediction of creatinine clearance (in mL/min) by the Cockcroft-Gault formula was calculated as (140 − age) × body weight/serum creatinine × 72 (× 0.85 if female).^23^ Based on previous study, CIN was defined as grade 0 (serum creatinine increase <25% above baseline and <0.5 mg/dL above baseline), grade 1 (serum creatinine increase ≥25% above baseline and <0.5 mg/dL above baseline), or grade 2 (serum creatinine increase ≥0.5 mg/dL above baseline).^18^

### Statistical Analysis

The continuous variables are expressed as mean ± standard deviation, and they were compared using the Student t-test or the Mann-Whitney U-test, as appropriate. The categorical variables are expressed as frequencies and percentages, and they were compared between the aforementioned groups applying the χ2 test or the Fisher exact test. All *P* values <0.05 were considered statistically significant. All the data analyses were conducted using SPSS (version 19.0) (Chicago, Illinois, US).

## Results

A total of 295 patients with ST-segment elevation myocardial infarction (STEMI) undergoing primary PCI were enrolled in the study. The patients were randomized to 80 mg atorvastatin (n = 144) or 40 mg rosuvastatin (n = 151), respectively, prior to primary PCI. The baseline characteristics were not difference between 2 groups (Table 1). Also, Mehran’s CIN risk score was not different between statin groups.

**Table 1:** Baseline characteristics of the 2 study groups

**Variables**	**Drugs**		***P*** ** value**
	**Rosuvastatin (n = 151)**	**Atorvastatin (n = 144)**	
Age > 75 years old, No. (%)	15(9.9%)	8(5.6%)	0.161
Male gender, No. (%)	130(86.1%)	118(81.9%)	0.330
Cigarette smoking, No. (%)	65(43.0%)	75(52.1%)	0.120
Diabetes mellitus, No. (%)	42(27.8%)	38(26.4%)	0.783
Hypertension, No. (%)	60(39.7%)	72(50.0%)	0.076
Hypercholesterolemia, No. (%)	8(5.3%)	14(9.7%)	0.148
Prior CABG, No. (%)	13(8.6%)	7(4.9%)	0.201
Prior PCI, No. (%)	25(16.6%)	18(12.5%)	0.324
Total cholesterol	166.4 ± 42.4	168.8 ± 42.0	0.638
Low-density lipoprotein	104.2 ± 76.2	102.0 ± 33.6	0.372
High-density lipoprotein	42.4 ± 8.9	40.7 ± 7.9	0.117
Triglycerides	123.6 ± 56.7	136.2 ± 77.0	0.342
Hemoglobin	14.5 ± 1.6	14.4 ± 1.5	0.567
Angiotensin-converting enzyme-inhibitor, No. (%)	103 (68.2%)	94 (65.3%)	0.593
Angiotensin II receptor blocker use, No. (%)	27 (17.9%)	33 (22.9%)	0.283
Beta-blocker use, No. (%)	118 (78.1%)	115 (79.9%)	0.718
Diuretic use, No. (%)	55 (36.4%)	50 (34.7%)	0.760
Calcium channel blocker, No. (%)	11 (7.3%)	11 (7.6%)	0.908
Angiography data, No. (%)			
Multi-vessel	85 (56.3%)	81 (56.3%)	0.994
Single-vessel	65 (43.0%)	61(42.4%)	0.905
Ejection fraction < 30%	36 (23.8%)	31(21.5%)	0.635
Mehran’s contrast-induced nephropathy risk score, No. (%)			
≤5	57 (37.7%)	58(40.3%)	0.966
6–10	67 (44.4%)	62(43.1%)	
11–16	23 (15.2%)	21(14.6%)	
≥ 16	4 (2.6%)	3(2.1%)	

**Figure 1:**
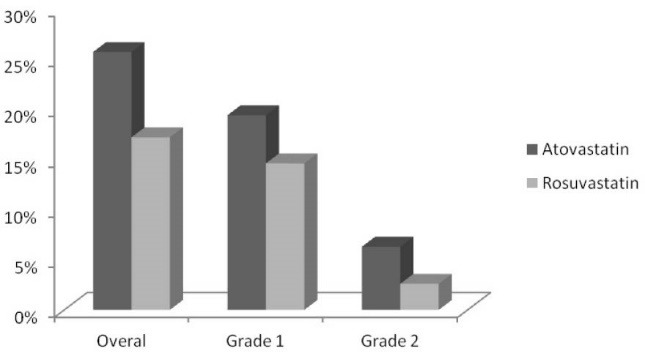
The frequency of CIN was not significant difference found between statin treatment groups.

Totally, 36 patients (12.2%) had eGFR lower than 60 mL/min/1.73 m^2^; which was not different between groups (*P* value: 0.36). After 48 hours from primary PCI, the CIN were observed in 63 patients (21.4%). Of those, 17% (n = 50) were grade 1 CIN, while 4.4% (n = 13) were grade 2 CIN. There was no significant between statin groups regarding the CIN grading (*P* value: 0.14) (Table 2; Figure 1).

**Table 2:** Baseline and 48 hour laboratory data and frequency of CIN between groups

	**Rosuvastatin (n=151)**	**Atorvastatin (n=144)**	***P*** ** value**
Baseline creatinine (mg/dL)	1.02±0.41	0.93±0.42	<0.001
Baseline BUN (mg/dL)	18.5±8.02	16.9±8.3	0.002
Baseline eGFR (mL/min/1.73 m^2^)	94.06±33.1	107±39.01	0.002
Baseline eGFR <60 mL/min/1.73 m^2^, No. (%)	21 (13.9)	15 (10.4)	0.362
48 hours creatinine (mg/dL)	1.08±0.54	1.03±0.57	0.009
48 hours BUN (mg/dL)	21.2±10.5	20.4±13.1	0.028
48 hours eGFR (ml/min/1.73 m^2^)	90.2±33.6	98.9±40.0	0.053
Creatinine, ∆ (from baseline to 48 hours) (mg/dL)	0.07±0.28	0.11±0.34	0.300
Contrast induced nephropathy, No. (%)			
Grade 0	125 (19.4)	107 (74.3)	0.144
Grade 1	22 (14.6)	28 (19.4)	
Grade 2	4 (2.6)	9 (6.3)	

## Discussion

The present study revealed that high doses rosuvastatin in setting of STEMI patients who underwent primary PCI on preventing CIN is effective compared with high dose atorvastatin. Muñoz et al^17^ compared the efficacy of simvastatin and pravastatin for CIN prophylaxis among 261 patients and reported rates of 17.9% and 8.6% in the simvastatin and pravastatin groups, respectively, with the difference constituting statistical significance. There was no dialysis-requiring case in their study, similar to our study. However, the authors found that 14.5% and 6.9% of their patients in the simvastatin and pravastatin groups, correspondingly, had acute renal failure - with a significant difference. Totally, they concluded that pravastatin had better efficacy for CIN prophylaxis.

Leoncini et al^16^ had 2 groups of patients with and without rosuvastatin and reported that 15.1% and 6.7% had CIN in the control and drugs groups, respectively, showing a statistically significant difference. In addition, their rosuvastatin group experienced lower death and re-infarction rates.

Toso et al^19^ compared 2 groups of patients with and without atorvastatin and reported that 11% in the control group and 10% in the drugs group had CIN, showing no statistically significant difference. The investigators concluded that atorvastatin had no effect on CIN prevention. Pappy et al^20^ revealed in their meta-analysis that statins were effective drugs for CIN prophylaxis, which is concordant with our results. The results of our study are reliable because of the use of group matching and reduction of the effects of confounding factors.

Kaya and colleagues^18^ for the first time compared high dose atorvastatin and rosuvastatin for CIN prophylaxis among 192 patients with STEMI under primary PCI and reported that totally 8.9% had CIN without significant difference. They showed that the grade 1 CIN occurred in 9.2% and 5.3% of patients treated with atorvastatin and rosuvastatin groups, while the rates for grade 2 CIN occurred 1% and 2.1%, respectively (*P* value: 0.50). Compared with our study, presence of CIN was more in our observation (12.2%); however, same as Kaya and colleagues^18^ there was no significant difference were observed between groups in our study.

Totally, according to the obtained results, it may be concluded that atorvastatin and rosuvastatin have similar efficacy for preventing CIN. However, further studies with larger sample sizes and multi-center samplings are required to attain more definite results.

## Ethical approval

Informed consent was received from all the patients, and the Helsinki Declaration was observed throughout the study. The local ethics committee of the center approved this study.

## Competing interests

None.
